# Assisted partner notification services for patients receiving HIV care and treatment in an HIV clinic in Nairobi, Kenya: a qualitative assessment of barriers and opportunities for scale‐up

**DOI:** 10.1002/jia2.25315

**Published:** 2019-07-19

**Authors:** Aliza Monroe‐Wise, Peter Maingi Mutiti, Harun Kimani, Hellen Moraa, David E Bukusi, Carey Farquhar

**Affiliations:** ^1^ Departments of Global Health and Medicine University of Washington Seattle WA USA; ^2^ Kenyatta National Hospital Voluntary Counseling and Testing Centre Nairobi Kenya; ^3^ Department of Community Health Kenyatta University Nairobi Kenya; ^4^ Department of Epidemiology University of Washington Seattle WA USA

**Keywords:** HIV testing, Kenya, assisted partner notification services, disclosure, barriers, facilitators

## Abstract

**Introduction:**

Identifying HIV‐positive individuals is increasingly recognized as one of the most important and most challenging of the UNAIDS 90‐90‐90 goals. Assisted partner notification services (aPNS) involves tracing and offering HIV testing to partners of HIV‐positive individuals, and is effective and safe when provided to newly diagnosed HIV‐positive patients. Voluntary aPNS is now part of the World Health Organization's guidelines for HIV prevention and care. However, uptake of aPNS is significantly lower among adults with established HIV infection already engaged in care compared to newly diagnosed individuals. We sought to describe barriers encountered and potential opportunities to providing aPNS to established patients living with HIV.

**Methods:**

We conducted focus group discussions and in‐depth interviews at Nairobi's largest public HIV clinic in April to May 2016 to elucidate barriers to and opportunities for aPNS among established patients engaged in HIV care. Participants included HIV‐positive adults in care, their partners, and healthcare workers (HCWs). Qualitative data analysis took a grounded theory approach.

**Results:**

Barriers to aPNS fell under three main categories. Fear of disclosure to partners included concerns over relationship repercussions, loss of trust, blame and violence. Stigma and discrimination were described in the healthcare setting, at church and in general society. Participants described difficulties approaching communication, including cultural barriers and differences in education. For almost every barrier a potential solution was also identified, and a barrier‐opportunity relationship emerged. Opportunities included using couples testing centres to aid in disclosure, focusing on the ambiguous introduction of the infection, and sensitization of HCWs and community leaders.

**Conclusions:**

aPNS among established HIV patients is associated with different barriers and opportunities than aPNS among newly diagnosed patients, and HCWs should build their capacity to support aPNS in this population. There is a strong need for increased training and sensitization on the use of aPNS in different circumstances and for different clients, taking into consideration factors such as timing of partner notification, characteristics of the relationship and duration of knowledge discordance. The overall success of this intervention among populations living with HIV may rely on customization of services and key messages to meet the patients’ specific needs.

## 1 Introduction

As countries throughout Africa strive to achieve the UNAIDS 90‐90‐90 goals by 2020 1, diagnosing 90% of those living with HIV remains the most elusive target worldwide 2, and strategies to reach high‐risk individuals with HIV testing have become increasingly important 3. According to UNAIDS 4, of the 36.9 million people living with HIV (PLHIV) in the world, over 9.2 million do not know their HIV‐positive status. Sexual partners of PLHIV in sub‐Saharan Africa have an HIV prevalence ranging from 35% to 72%, over seven times that of the general population 5. In Kenya, slightly more than half (53.1%) of HIV‐positive individuals were not aware of their status in 2014 6, and HIV prevalence among partners of HIV‐positive individuals is over 20% 7, compared with the HIV prevalence in the general population of 5.9% 8.

Assisted partner notification services (aPNS) significantly and safely increases the uptake of HIV testing services (HTS) for partners of newly diagnosed PLHIV and can improve case‐finding and linkage to care 5. aPNS is now part of the World Health Organizations’ guidelines for standard practices for PLHIV 9. This strategy has been successfully implemented among newly diagnosed PLHIV in the USA 10, Mozambique 11 Malawi 12, 13 Tanzania 14, Cameroon 15 and Kenya 7 among others, and has recently been adapted for a scale‐up study in the Comprehensive Care Centre (CCC – HIV care clinic) at Kenya's national referral hospital, Kenyatta National Hospital (KNH). However, the uptake of aPNS among established CCC clients (i.e. those who have started antiretroviral therapy) at the KNH CCC has been noted to be significantly lower than that in trials among newly diagnosed PLHIV, with only 52% of clients enrolled in care at the KNH CCC accepting aPNS [H. Kimani, unpublished data]. Although the risk of transmission from established patients on antiretrovirals (ARVs) to their partners is generally low due to viral suppression, the partners of HIV‐positive individuals have a high prevalence of HIV and are often unaware of their status 7, making them an important population for prioritized, targeted HIV testing interventions.

There are numerous barriers to undergoing HTS 16, including fear, lack of knowledge, perception of risk, relationship attributes, healthcare system characteristics and testing location 17, 18, 19, 20, 21. Similarly, while uptake of aPNS among newly diagnosed clients is generally high 7, those that refuse may do so due to a need for time to process the diagnosis, lack of trust in healthcare workers (HCWs), and misunderstanding of the process of aPNS 22. However, very few studies have explored barriers or opportunities for aPNS among PLHIV who are established patients at a CCC, although these individuals refuse aPNS more frequently and may have very different experiences of aPNS than those who are newly diagnosed. The purpose of this study was therefore to identify opportunities and barriers to aPNS among established clients at KNH's CCC.

## 2 Methods

### 2.1 Study design and setting

We performed a nested qualitative study within a randomized clinical trial conducted at the KNH CCC. KNH is the largest national teaching and referral hospital in Nairobi, Kenya. The KNH CCC currently has over 7000 active clients. Individuals are referred to the KNH CCC from clinics and wards within the hospital, in addition to facilities all over the city of Nairobi. As one of the first centres to offer treatment for Nairobi's PLHIV, many patients at the KNH CCC have been on ARVs for over a decade.

### 2.2 Parent study

Conducted throughout 2015, the parent study aimed to compare aPNS to passive notification among established patients at the KNH CCC. The study enrolled male and female HIV‐positive clients presenting for routine care in the CCC over a year's time as index participants, then randomized the index participants to either passive referral or aPNS. However, a significant number (48%) of participants randomized to the aPNS arm provided false contact information for their partners, which was interpreted as a way to opt out of aPNS in that arm.

### 2.3 Participant selection

Selective purposive sampling was used to recruit participants at the CCC (Table 1). Participants for focus group discussions (FGDs) included 7 to 10 men and women, and were drawn from individuals presenting to care during the study timeframe who met different criteria for three groups: existing KNH CCC clients in care, regardless of whether they had accepted or declined aPNS (three groups); partners of existing CCC clients in care who had utilized aPNS (two groups); and HCW working at the clinic (one group). HCWs were purposively sampled to include one individual from each different cadre of providers including clinical officers, nurses, nutritionists, HTS providers, peer mentors and a records officer. HCWs were selected on the basis of their availability during the time specified for the FGD. Participant selection for in‐depth interviews (IDI) was designed to purposively enrol equal numbers of men and women, and were drawn from index CCC patients in care, regardless of whether they had accepted or declined aPNS (five IDIs), and partners of index CCC clients who had utilized aPNS (five IDIs).

**Table 1:** Stratified purposive sampling framework

In‐depth interviews		Focus groups (each comprising 7 to 10 individuals)		
Index clients registered at KNH CCC	Partners of index clients at CCC	Index clients registered at KNH CCC	Partners of index clients at CCC	Healthcare workers at KNH CCC
No participants = 5	No participants = 5	No of groups = 3	No of groups = 2	No of groups = 1
Aim for range of age and sex	Aim for range of age and sex	Aim for range of age and sex	Aim for range of age and sex	Aim for range of age and sex

CCC, Comprehensive Care Centre; KNH, Kenyatta National Hospital.

### 2.4 Human subjects protections

All participants provided written informed consent. Data collection took place in a private location within the clinic, and staff were trained in confidentiality measures. Data were de‐linked from identifiers and kept in password‐protected files. Counsellors at the CCC were trained to respond to emotional distress relating to HIV testing procedures. This study was nested within a parent trial that was approved by the KNH/University of Nairobi Ethical Review Committee (KNH/UoN ERC, Protocol #P281/05/2015).

### 2.5 Study procedures

Six FGDs and 10 IDIs were conducted in Kiswahili and English by a trained Kenyan researcher experienced in qualitative data collection. Data collection took place in April to May, 2016, and each lasted between 60 and 90 minutes. Participants were reimbursed for their time and transportation. The interviewer used semi‐structured guides, and recorded audio data using a digital recorder with the participants’ consent, in addition to taking detailed notes. Guides were developed based on *a priori* research aims combined with preliminary informal responses from index clients at the CCC who refused uptake of aPNS services in the parent study, and the first IDI and FGD were used to pilot the guides. Guides for IDIs and FGDs differed in format but not in content. Minor changes to the guides were made prior to ongoing data collection for clarification of questions, and both pilots were included in the data. The topics covered in the IDIs and FGDs included index and partner experiences with aPNS services, understanding of aPNS services for both the clients and HCWs, and experiences including challenges and success stories in providing the services. All planned IDIs and FGDs were completed even after saturation was achieved. Saturation was defined as the point after which new data did not generate new themes during analysis. The same researcher transcribed IDIs and FGDs in their original languages (mostly Kiswahili, with occasional use of tribal languages), translated them into English, and back translated them to verify an accurate translation.

### 2.6 Data analysis

The analysis process was inductive and took a grounded theory approach 23. Transcripts were analysed by two coders: the investigator and the interviewer. Analysis began with open coding, which was followed by axial coding. Themes emerged and were organized in two main central categories: barriers to and opportunities for aPNS. The coders independently analysed all the transcripts and added additional salient themes to the code list using open coding. The interviewer compiled the two code lists to create a master codebook, and the two coders discussed the codebook to come to an agreement about each code. Thematic relationships between categories were identified, and theoretical relationships between barriers and opportunities emerged. ATLAS.ti version 7.5.7 (Berlin, Germany) was used by all the coders. After coding, the two coders selected quotes that best represented each theme and subtheme. The quotes that were selected by both coders were included as representative.

## 3 Results

A total of 47 participants, including index clients, partners and HCWs in the clinic, completed the study (Table 2). Thirty‐seven of these were included in FGDs and 10 completed IDIs. Thirty‐one of the 47 participants were female, and 33 of the 38 client participants were HIV positive. Four of the fourteen partners (28.6%) were HIV‐negative, representing serodiscordant couples, and one had unknown status.

**Table 2:** Participant characteristics for FGDs and IDIs

	No.	Sex		Age	Marital status					Education			HIV status		
		F	M	Mean (range)	Married monog.	Married polyg.	Single or dating	Widowed	Divorced/separated	None or primary	Secondary	Tertiary	Negative	Positive	Unknown
FGD (6)															
Index 1	7	5	2	46.1 (28, 72)	3		1	1	2	1	1	5		7	
Index 2	7	6	1	48.7 (33, 65)	4			1	2	2	3	2		7	
Index 3	5	5	0	41.1 (30, 53)	3		1		1	1	3	1		5	
Partners 1	5	3	2	44.4 (28, 55)	3			1	1	3	2		2	3	
Partners 2	4	2	2	37.5 (29, 48)	1		1			1	3			4	
HCWs	9	4	5												9
IDI (10)															
Index	5	4	1	34.6 (30, 42)	2		1		2	1	3	1		5	
Partner	5	2	3	41.0 (29, 50)	4	1				2	3		2	2	1

FGDs, focus group discussions; HCWs, healthcare workers; IDIs, in‐depth interviews.

**Figure 1:**
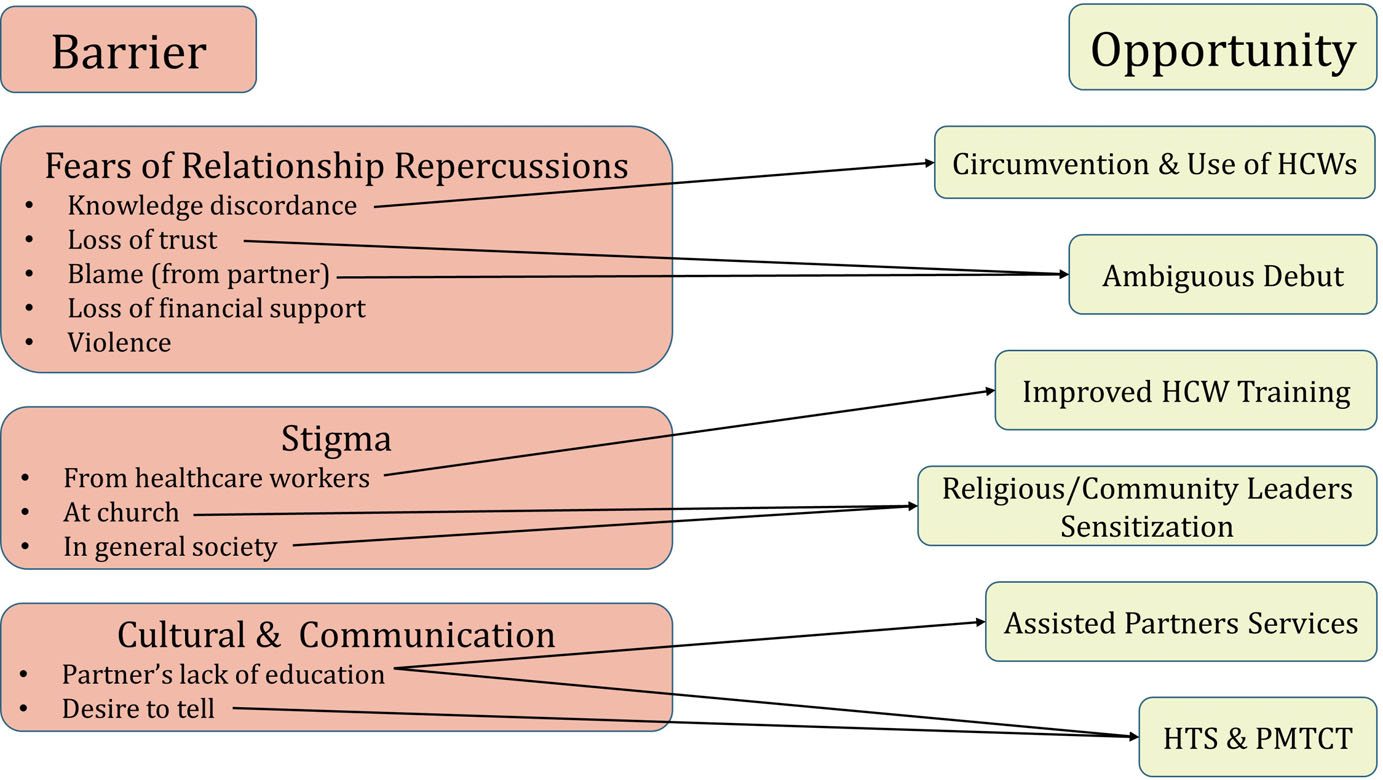
**The barrier‐opportunity relationship**HCW, healthcare workers; HTS, HIV testing services; PMTCT, prevention of mother‐to‐child transmission.

Throughout discussions, barriers to aPNS were mentioned alongside opportunities perceived to address those barriers. As certain participants discussed their fears surrounding aPNS, others told stories of how they had overcome the same fearful situations. As such, a “barrier‐opportunity continuum” emerged in which almost all barriers mentioned were associated with corresponding opportunities (Figure 1). Three main categories of barriers emerged, and were defined as fears of relationship repercussions, stigma and discrimination, and cultural and communication barriers.

Almost every barrier mentioned was based on a fear that undergoing aPNS would result in disclosure of the index client's status, either to the partner or to other entities, such as the church. Although aPNS has been associated with very few adverse events when previously studied 5, 25, the participants’ fear of disclosure was strong and sufficient enough to constitute a basis for the majority of barriers mentioned. As a result, the barriers discussed are not directly related to aPNS procedures, but are rather linked to the overarching fear of disclosure. This is significant, as it implies that a better understanding of how aPNS can protect the index client's identity and HIV status may alleviate many of the fears and barriers to aPNS cited by participants in the study.

### 3.1 Fears of relationship repercussions

The most commonly cited barriers to undergoing aPNS related to fears of repercussions in the relationship. These fears took many different forms and ranged in severity from a sense of possible conflict to fear of violence or relationship dissolution. Additionally, several individuals shared stories of actual dissolution of relationship, lending credence to stated fears.

On one end of the spectrum were those individuals with fears of creating conflict in the relationship. This fear was stated as a barrier, although this was not considered insurmountable, leading to the possibility of working with the couple to overcome any possible conflict that might arise from disclosure:

The biggest thing is fear, how will she take it [and] how will she react and what will be the extent of the reaction. Will my partner accept me or no. That is why being told at the hospital is a bit easy for your partner to tell you. (Partner FGD 1: *Male, 47 years*.)

Inherent in the above quote is both the stated barrier and the associated opportunity of HCW‐assisted notification. “Being told at the hospital is a bit easy,” the participant states, implying that those with general fears about notifying a partner might be assuaged in a healthcare setting, with the proper counselling.

The relationship is paramount in these discussions, and supersedes all other factors in making a decision to undergo aPNS. While “being told at the hospital is a bit easy,” the participants indicate that what happens in the relationship after disclosure has taken place may still be devastating. It is therefore of utmost importance that healthcare providers offering aPNS receive training in both discussing the low risk of adverse events associated with aPNS, and in couples counselling and relationship guidance in the setting of aPNS‐related disclosure.

#### 3.1.1 Knowledge discordance

One of the frequently cited perceived barriers in this population was the fear of one's partner discovering the presence of a “knowledge discordance,” or difference between the index partner's knowledge of his/her status as compared with the partner. In these examples, the actuality of the possible serodiscordance was less important than the discovery that the index partner had been tested alone.

Circumventive HCW‐assisted disclosure through the use of a Voluntary Counseling and Testing (VCT) or Couple HTS (CHTS) was described as a possible solution to the problem of knowledge discordance. For instance, several people described bringing their partners to get tested in a CHTS without letting on that they had previously been tested. In these cases, even the discovery of serodiscordance seems to be less of a burden when the couple is tested together:

If I happen to get a boyfriend, I would tell him to come with me to the couple counseling. [I] am sure they will be able to handle it better than me telling him, “this is my status, go and have your test done.” So it's better he gets tested while I get retested, then I see his reaction. If he doesn't accept me, I would rather move on without him. I would rather we go together than doing it individually. (Index FGD 3: *Female, 30 years*.)

This quote also illustrates the important role that healthcare providers can play in counselling couples to decrease potential mistrust and anger that arises when couples learn of serodiscordance, and in supporting their relationship throughout the testing process.

Circumvention as an opportunity to alleviate knowledge discordance was also mentioned in a different context. Here, circumvention is employed by an index client invoking a fictional requirement from one's workplace that partners get tested:

He told me “At work we were told that we have to take our wives to Kenyatta for testing and the results are needed at work.” (Partner FGD 1: *Female, 44 years*.)

The timing of disclosure was stressed by some participants as an important aspect in pre‐empting possible discomfort and strife commonly associated with knowledge discordance, with early disclosure far better than late. In disclosing early, the participants were relieved of the burden of the discordance before it grew into a more significantly toxic element of the possible disclosure event.

I feel if … I started dating somebody, the first thing I would do is to tell that person. I would disclose to that person so that if they opt to stay with me, they do so knowing and if he is to leave then he just leaves. Because I think it's very, very important to protect somebody psychologically. (Index FGD 3: *Female, 53 years*.)

#### 3.1.2 Assigning blame

Throughout the interviews and focus groups, a recurrent theme was the fear of being blamed for one's HIV status. This potential blame included being blamed for infidelity, and losing the trust of one's partner. Discussions and speculation surrounding who introduced the infection into the relationship were pervasive, and theoretical blame was assigned for this potential transgression:

I was to blame my wife but I said no let me not blame her according to how doctors were explaining it to me. (Partner IDI: *Male, Partner 45 years*.)

Interestingly, while discussing the idea of blame, participants were also aware of an inherent inability to definitively identify the person or moment of the initial infection. Participants reflected back on decades of their sexual histories in an attempt to identify the possible initial infection:

At the same time I did not want to say he was the one who had infected me. I was asking myself, “before I met him, didn't I date?” I remembered that I had dated another policeman, who I later on heard that he died. So, even if I was to say it was this one, what about the other one? (Partners FGD 2: *Female, 29 years*.)

This idea of the “ambiguous debut,” or the impossibility of knowing with any certainty when or how the infection was introduced in a relationship was a source of comfort to many participants, whether they were in serodiscordant or seroconcordant relationships. In seroconcordant relationships, the ambiguity of the debut was related to which person may have first acquired HIV, as in the quote above. The ambiguity of the debut in serodiscordant relationships surrounded the timeframe in which the HIV‐positive partner might have acquired the infection:

She would have blamed me, but it is even hard to tell when you got infected. (Index FGD 2: *Male 52 years*.)

Two separate themes were apparent in discussions of how HCWs might help with blame and mistrust as a result of HIV status disclosure. First, as stated above, HCWs might mitigate relationship discord that can arise as a result of undergoing testing:

There is that guilt among two [people]: “Oh it is you who caused it, it is you who did.” But at the hospital … there is that counseling that can help someone … handle that situation better. (Partners FGD 1: *Male, 47 years*.)

The other way in which participants believed HCWs could be helpful to couples undergoing testing was that they believed their partners might pay more attention to the advice of HCWs, inciting them to take action whereas the index participant's urging could not:

I think he should be called in such a way that he can easily come. On my part if I keep telling him to come, he won't agree … So, I have asked another counselor to assist me, because I really want him to get help. (Index FGD 3: *Female, 44 years*.)

#### 3.1.3 Fear of loss of financial support

Several participants expressed such profound fear of creating conflict in their relationship as a result of possible serodiscordance that they stated they would rather infect their partners than discover that their partners were negative while they were positive. This extreme fear was linked to financial dependence on the partner with unknown status, and was typically reported by a female about a male partner.

Other challenges are due to dependency, for example if you are a wife and you depend on your husband to provide your needs. In that case you will not tell your husband because if he abandons you, it will be hard for you to meet your needs; maybe you also have children who depend on you. [Because of] the fear that if I tell him and he abandons me, I would rather infect him so that we can deal with it later. (Index FGD 2: *Female 43 years*.)

#### 3.1.4 Fear of violence

Several participants reported fearing a violent reaction from their partners. These reactions ranged from kicking and punching to murder.

It could be anything. He could commit suicide, he could kill me, kick me out or part ways with me. (Partners FGD 2: *Female, 27 years*.)

Additionally, several participants reported they feared that they themselves may take violent actions, either towards a partner or towards themselves.

So when I am alone I say that even I can throw myself from the upper floors of the house and die because I used to see those people that have it the way they grow thin and they look bad. One day he brought himself [to] Kenyatta and he … tested and from that time he knew. [But] because I always used to tell him that I will hang myself, he was afraid of telling me. (Partners FGD 1: *Female, 44 years*.)

Healthcare‐worker assisted notification was cited as a mitigating factor for violent responses.

The advantage of this method, [of] assisted disclosure is that it has really helped to mitigate injuries or violence because when this kind of a thing happens in the house, we don't have neutral ground. Of course in that process of acceptance … bad things can happen, maybe suicide, domestic violence. So it has really helped to mitigate those kind of things. (HCW FGD: *Male Clinical officer*.)

### 3.2 Stigma and discrimination

Societal stigma related to HIV remained a dominant concern among participants, and appeared to affect most individuals’ decision to disclose their status. Participants feared stigma coming from many aspects of their lives, including family, HCWs, church and the workplace.

#### 3.2.1 From HCWs

Stigma and blame from HCWs is particularly concerning, considering the important role that HCWs play in the diagnosis, linkage and maintenance of care for HIV‐positive individuals. This was discussed not only in a hypothetical anticipatory sense, but was also mentioned by several participants when describing the actual events that occurred when they or their partners were tested.

In counseling my wife, the nurse was blaming me, that I could have infected my wife. And so when being blamed [I knew that] it was not like that since you understand yourself. So, I had to do my test, and I was negative … (Partners FGD 1: *Male, 47 years*.)

Additionally, several participants described seeking medical advice for illness when medical practitioners were not straightforward about the possibility of HIV infection. In the following example, the participant sought the opinion of a HCW, who told him that he had a “nutrition” problem.

When I came for testing for the first time, I realized that my health was deteriorating. I had already visited various physicians, three of them to be precise, before I came here. The last one told me that I was not eating enough food, so he suggested that I leave and go have enough food. I asked him if he was offering HIV testing and he said no; however, he pointed out that a blood sample could be taken from a patient and taken to another place … for analysis and the results would be brought later. (Index FGD 2: *Male, 56 years*.)

In this example, the reluctance of the healthcare provider to discuss the realities of HIV risk, diagnosis and treatment may be associated with the stigma surrounding infection, and can contribute to barriers to an individual getting tested and treated.

#### 3.2.2 In general society

In addition to HCWs, participants described fear of stigma from many other sources. Several people felt general society would stigmatize an individual known to be infected with HIV.

[If found to be infected], you will lack friends, they will avoid you completely, they will start to back bite you and talk ill about you (Partner IDI: *Female, 29 years*.)

Conversely, participants cited several other sectors of society as potential factors in motivating individuals towards HIV testing and disclosure. First, many people mentioned family members as potential agents of change.

In 2006 my elder sister called me to her place. She told me, “my sister your sickness has become too much. Whatever has happened has happened. Let's just go, there is a health center [nearby] so that we can see what is going on.” I told her I could not … She persuaded me and I agreed. (Partners FGD: *Female, 48 years*.)

In this quote, what is striking is that the participant's sister did not just urge the participant to get tested, but actually went with her to the health centre to seek services. In this case, it is not just the discussion but the physical presence of a trusted family member that helped the participant overcome her barriers.

#### 3.2.3 At church

Church was also mentioned as a potentially stigmatizing institution, with several people citing specific examples of how the leadership of their churches might discriminate against them if they were aware of the participant's HIV status.

Even within the church you cannot be given a role, you are discriminated against. They can't even allow you to cook, they can only ask you to fetch water and bring firewood. (Index FGD 2: *Female, 43 years*.)

Ongoing efforts to sensitize societal leaders, including religious and community leaders, might be a potential opportunity to redouble efforts to address the fear of stigma experienced by PLHIV and those undergoing HTS in the future.

### 3.3 Cultural and communication barriers

Several participants cited barriers related to the mechanics of navigating cultural expectations and mores, and the process of actually communicating their status to their partners.

#### 3.3.1 Partner's lack of education

One participant described her partner as being very “soft,” or unsophisticated, due to his upbringing in a rural area and lack of education. Due to his belief in witchcraft and misunderstandings surrounding HIV, the participant reported discomfort with the idea of disclosing her status.

I am dating a very soft guy and from a humble background where people still believe in witchcraft and they do not want to believe there is HIV. And then if you happen to be positive, you are discriminated against, when you are walking around, people will be talking, you become the story. So if you are dating somebody from that place, and you want to tell him that you are positive, how will he view you? How will he be viewed in the [community]? That is what will come to his mind and he will say, “Let me kill this fool and kill myself.” (Partners FGD 2: *Female, 29 years*.)

#### 3.3.2 Desire to tell

There were several participants who reported having a strong desire to disclose to the partner, but lacking a plan with respect to the timing and wording of the disclosure.

I really desire to tell him but I wonder what will he think, how will he say. Deep down, I desire to tell him and I have tried thrice though from afar. I fail to tell him and he suffers while I know and he doesn't know … The only thing that is remaining is if I could get a way of disclosing this thing safely. Something is undone, and I don't know how to get it done. I will keep my medicines where he can see them but he doesn't know what they are for. But I am unable to tell him … (Partners FGD 2: *Female, 29 years*.)

Training HCWs to discuss aPNS as a means of disclosure could help individuals who have expressed a desire to disclose move forward with notification, removing barriers their clients experience.

## 4 Discussion

We conducted a qualitative assessment of barriers and opportunities to aPNS after finding low uptake of aPNS among established patients at Kenya's largest CCC, and found differences and similarities between barriers and opportunities for aPNS among established clients receiving care at the KNH CCC and those identified in research among newly diagnosed individuals. Goyette, et al. conducted a qualitative study of barriers to aPNS in 2016 22, which found that many people were still grappling with their own diagnosis and mentioned that they were “shocked,” “in denial,” and “did not want to talk to anyone” after learning of their diagnosis. None of the participants in our study appeared to be struggling with accepting their own diagnosis, and none mentioned reactions such as these. Trust between clients and HCWs was also identified as a major barrier for newly diagnosed PLHIV, who were more likely to engage in aPNS if they were shown empathy or felt connected to the HCW who diagnosed them 22. In our study, although some criticism of HCWs was discussed, lack of trust in HCWs was rarely mentioned. This may reflect the positive impact of long‐term patients’ relationships with healthcare providers at their clinics in this population of established patients. A further unique barrier identified in our study among established patients was the fear of disclosing one's status in the setting of having carried the diagnosis for a long period of time without the partner's knowledge (knowledge discordance). This and the related issue of the timing of disclosure were cited as powerful factors affecting the decision to undergo aPNS in our study.

Our study also found several similarities with previous studies of barriers to aPNS among newly diagnosed clients. Most barriers mentioned in our population were related to the fear of disclosure of one's status to his or her partner and the relationships repercussions this might cause. These fears were similar to fears expressed in research among newly diagnosed PLHIV. Studies have demonstrated that sex and partner type play important roles in barriers to aPNS for index participants 25. While our study was not designed to investigate these differences, we did note a gender dynamic among women discussing fear of the loss of financial support. This indicates that one significant factor behind female reluctance to undergo aPNS is many women's ongoing financial reliance on male partners.

Previous research has identified several strategies that can be used to support newly diagnosed PLHIV in disclosing their status to partners. In some cases, passive notification was preferred, as patients felt their relationships to partners was stronger and more important than relationships with HCWs in the clinics 26. In one study of public health practices, barriers to aPNS included indexes’ reluctance to share partner information with providers 27. In this case, separate trained notification specialists were employed in an attempt to mitigate the lack of relationship between newly diagnosed PLHIV and providers. aPNS in a limited, controlled research setting might result in higher acceptance rates than that in public health practice.

Through grounded theory analysis, our study has identified a theory that most barriers exist on a continuum with an associated opportunity for improvement. Potential solutions to the problems identified in our data were often different from those employed for use among newly diagnosed clients undergoing aPNS. One commonly cited opportunity among our participants was the use of circumventive HCW‐assisted disclosure at a CHTS clinic as described by our participants to overcome the issue of knowledge discordance. Other solutions included emphasizing the ambiguous debut, sensitization of HCWs and community leaders, and education surrounding the practice of aPNS. The interpretation of our data is limited by several factors. First, it is possible that our sample is not representative of the general population at an HIV clinic. Notably, there were only four HIV‐negative partners included of the 13 enrolled, indicating that the majority of couples represented in the sample were seroconcordant. This may represent the prevalence of seroconcordance at the CCC, where many patients have been living with HIV for many years. Additionally, we had an uneven number of men and women in our sample, with women outnumbering men almost 2 to 1. This may be a reflection of the larger proportion of women than men at the CCC. Our sample also represented individuals who had been enrolled in care for years, and may not accurately represent HIV‐positive individuals in other settings. Finally, many of our participants accepted aPNS, and therefore may not represent individuals who are limited by the perceived associated barriers. Nevertheless, our sample does represent the experiences and opinions of HIV‐positive individuals engaged in care at one of Nairobi's largest CCCs who have faced the decision to undergo aPNS.

## 5 Conclusions

aPNS can be a powerful tool to identify and test individuals at high risk for HIV, and is now part of the World Health Organization's testing guidelines 9. However, providers offering aPNS should be aware of pitfalls and opportunities inherent in conducting notification, particularly with respect to different types of clients one might encounter. These pitfalls must be identified and addressed prior to scale‐up efforts. Established clients face unique barriers to aPNS, but best aPNS practices for this population are not yet established. Circumventive referrals to CHTS clinics should become standard procedure for any index participant struggling with barriers to undergoing aPNS, and all HCWs in CCCs and CHTSs should be trained in this procedure. Furthermore, HCWs should receive training to understand the issue of knowledge discordance, and how to discuss ambiguous debut with both index clients and partners. Similarly, HCWs must be able to counsel clients on specific steps to take and language to use in the course of HIV status disclosure to overcome communication barriers. Packaging these skills into a training programme for all HCWs who care for PLHIV would allow scalability of the intervention. There also remains a strong need for community sensitization activities mediated by the national HIV prevention programme to provide both a supportive framework for and a basic knowledge of aPNS.

## Competing interests

All authors declare that they have no conflicts of interest.

## Authors’ contributions

PM, HK, DB and CF designed the research study. PM and HM collected the data. PM, HM and AMW analysed the data. PM and AMW wrote the paper. AMW, PM, HK, HM, DB and CF edited the paper, provided valuable critique and approved the final draft.
